# Effects of respiratory disease on Kele piglets lung microbiome, assessed through 16S rRNA sequencing

**DOI:** 10.14202/vetworld.2020.1970-1981

**Published:** 2020-09-25

**Authors:** Jing Zhang, Kaizhi Shi, Jing Wang, Xiong Zhang, Chunping Zhao, Chunlin Du, Linxin Zhang

**Affiliations:** Key Laboratory of Livestock and Poultry Major Epidemic Disease Monitoring and Prevention , Institute of Animal Husbandry and Veterinary Science, Guizhou Academy of Agricultural Sciences, Guiyang, Guizhou, China

**Keywords:** 16S rRNA sequencing, Kele piglets, microbial diversity, respiratory diseases, *Ureaplasma*, *Ureaplasma diversum*

## Abstract

**Background and Aim:**

Due to the incomplete development of the immune system in immature piglets, the respiratory tract is susceptible to invasion by numerous pathogens that cause a range of potential respiratory diseases. However, few studies have reported the changes in pig lung microorganisms during respiratory infection. Therefore, we aimed to explore the differences in lung environmental microorganisms between healthy piglets and piglets with respiratory diseases.

**Materials and Methods:**

Histopathological changes in lung sections were observed in both diseased and healthy pigs. Changes in the composition and abundance of microbiomes in alveolar lavage fluid from eleven 4-week-old Chinese Kele piglets (three clinically healthy and eight diseased) were studied by IonS5™ XL sequencing of the bacterial16S rRNA genes.

**Results:**

Histopathological sections showed that diseased pigs displayed more lung lesions than healthy pigs. Diseased piglets harbored lower bacterial operational taxonomic units, α-diversity, and bacterial community complexity in comparison to healthy piglets. Taxonomic composition analysis showed that in the diseased piglets, the majority of flora was composed of *Ureaplasma*, *Mycoplasma*, and *Actinobacillus*; while *Actinobacillus*, *Sphingomonas*, and *Stenotrophomonas* were dominant in the control group. The abundance of *Ureaplasma* was significantly higher in ill piglets (p<0.05), and the phylogenetic tree indicated that *Ureaplasma* was clustered in *Ureaplasma diversum*, a conditional pathogen that has the potential to affect the swine respiratory system.

**Conclusion:**

The results of this study show that the microbial species and structure of piglets’ lungs were changed during respiratory tract infection. The finding of *Ureaplasma* suggested that besides known pathogens such as *Mycoplasma* and *Actinobacillus*, unknown pathogens can exist in the respiratory system of diseased pigs and provide a potential basis for clinical treatment.

## Introduction

The swine respiratory tract is an important system that connects the external environment to the lower respiratory tract. After birth, microorganisms enter the lungs of pigs through the upper respiratory tract and influence respiratory activity. Changes in microbial diversity and a loss of host immunity lead to primary and opportunistic pathogen invasion and disease occurrence [1]. Swine respiratory disease is a complex disease caused by the synthetic action of various bacteria, viruses, *Mycoplasma*, and a loss of bacterial resistance [2]. Swine respiratory disease can be caused by various microbial pathogens, including *Mycoplasma hyopneumoniae* [3], *Pasteurella multocida* [4], *Salmonella enterica* serovar Choleraesuis [5], *Streptococcus suis* [6,7], *Actinobacillus pleuropneumoniae* [8], and *Haemophilus parasuis* [9]. The currently available vaccinations and antibiotic treatments are aimed at known bacteria associated with respiratory tract diseases of piglets, but have poor efficacy, possibly due to the involvement of unknown bacterial pathogens. It is, therefore, essential to guide the prevention and control of swine respiratory diseases through the clarification and identification of the microbial communities involved.

At present, the methods for the identification of specific microorganisms related to the pig respiratory tract include culture, single/multiplex polymerase chain reaction (PCR), and fluorescence quantitative PCR. However, these methods have numerous drawbacks. Producing reliable data from the culture method is time-consuming, and many respiratory pathogens are difficult to culture (such as *Mycoplasma*). Although the sensitivity of the PCR method is relatively high, it targets only known pathogenic bacteria [10,11]. High-throughput sequencing technology is a culture-independent, time-saving method to identify known and unknown microorganisms and is, therefore, a key and reasonable method for studying the microbial community in lungs in both healthy and diseased states [12-14]. The previous studies using high-throughput sequencing have identified all microbial communities in a range of samples, including clinically isolated strains [15,16], the human respiratory tract [17,18], the rumen of cattle [19], the bovine respiratory tract [20,21], and pig intestines [22,23]. In pigs, DNA shotgun metagenome sequencing has been used to sequence alveolar lavage fluid pools, providing a wide view of the bacterial populations of lungs with enzootic pneumonia and lungs lacking the signs of enzootic pneumonia in field situations. The most common microorganism families in the lungs of pigs with endemic pneumonia were *Mycoplasmataceae*, *Flavobacteriaceae*, and *Pasteurellaceae* [24]. In another study, 20 alveolar lavage fluid samples from 9 male and 11 female pigs artificially immunized with seven common vaccines were sequenced by 16S rRNA amplicon sequencing, and it was reported that reduced microbial diversity but higher biomass occurred in lungs with severe lesions (based on lung injury degree scores). *Methylotenera*, *Prevotella*, *Sphingobium*, and *Lactobacillus* were the main bacteria in healthy or slight lesion lung groups, while *Mycoplasma*, *Ureaplasma, Sphingobium, Haemophilus*, and *Phyllobacterium* were the most abundant microbes in severe lesion lungs [25]. Few studies have reported on the microbial diversity or lung changes in Kele piglets under natural infection or used high-throughput sequencing to provide a basis for clinical treatment.

Therefore, in this study, microbial diversity, community structure, and core microbes in the lungs of Kele piglets were analyzed and compared by 16S rRNA amplicon sequencing to explore the differences in lung environmental microorganisms between healthy piglets and piglets with respiratory diseases.

## Materials and Methods

### Ethical approval

The study protocol was approved by Animal Ethics Committee of the Institute of Animal Husbandry and Veterinary Science, Guizhou Academy of Agricultural Sciences (Guizhou, China), and the Laboratory Animal Center of Guizhou Firstv Biological Technology Co., Ltd. (approval No. SYXK-2015-001).

### Animals and sample collection

Eleven 4-week-old Kele piglets were obtained from Guizhou Province of China in December 2018. Eight piglets were clinically characterized as exhibiting respiratory disease (panting, reduced food intake, and reduced body growth). Three healthy piglets were also obtained locally. All piglets were euthanized (using narcotic drugs for general anesthesia and then let blood) and dissected. The pathological regions of lung tissue and other organs were observed in the diseased piglets. For both the diseased and healthy piglets, the whole lung was placed on ice and sent to the laboratory. The surface of each lung was flushed with sterile 1% phosphate-buffered saline (PBS) (Solarbo, Shanghai, China); then, the lungs were placed in a biological safety cabinet, and the lung alveoli were flushed with 50 mL of sterile 1% PBS. The alveolar fluid process was repeated 3-5 times. The alveolar lavage fluid was filtered through four layers of sterile gauze to remove debris, and this filtration process was repeated 3 times. The resulting fluid was centrifuged at 4000 × *g*, at 4°C for 30 min, and the supernatant was discarded. The remaining pellet was resuspended in 5 mL sterile PBS and stored at −80°C until further use.

### DNA extraction and PCR amplification

DNA was extracted from each lung using the phenol-chloroform method [26]. The DNA concentration and purity were monitored using 1% agarose gels. Based on the specific concentrations that were observed, the DNA in each sample was diluted to 1 ng/μL in sterile water. 16S rRNA genes from distinct regions (V3-V4) were amplified using a specific primer (338F-806R). The PCR product was detected by electrophoresis using 2% agarose gels. The PCR product was recovered using a Thermo GeneJET Gel Recovery Kit (Thermo Scientific, Massachusetts, USA).

### Library construction and sequencing

Eleven sequencing libraries from experimental piglets were generated using the Ion Plus Fragment Library Kit, 48 rxns (Thermo Scientific, Massachusetts, USA) following the manufacturer’s recommendations. The library quality was assessed using a Qubit@ 2.0 Fluorometer (Thermo Scientific, Massachusetts, USA). The library was sequenced using an Ion S5™ XL platform (Thermo Scientific, Massachusetts, USA) and 400 bp/600 bp single-end reads were generated.

### Sequencing data processing

Quality filtering on the raw reads was performed using specific filtering conditions to obtain high-quality, clean reads based on the Cutadapt quality controlled process [27]. The reads were compared with the reference database using the UCHIME algorithm to detect chimera sequences, and then, the chimera sequences were removed [28], which allowed the final clean reads to be obtained (NCBI accession number: PRJNA576744).

### Operational taxonomic unit (OTU) clustering and species annotation

Sequence analyses were performed using Uparse software [29], and sequences with ≥97% similarity were assigned to the same OTUs. Representative sequences for each OTU were screened for further annotation. For each representative sequence, the Silva database was used, based on the Mothur algorithm, to annotate the taxonomic information [30]. Taxonomic information was obtained for each sample, and the statistics of the community composition of each sample was obtained at each classification level: Phylum, class, order, family, genus, and species. Finally, the OTU abundance information was normalized using a standard of the sequence number that corresponded to the sample with the smallest number of sequences. Subsequent analysis of the alpha diversity and beta diversity was performed based on the normalized output data.

### Alpha diversity (α-diversity)

Alpha diversity was applied to analyze the complexity of species diversity for a given sample using six indices: Observed_species, Chao1, Shannon, Simpson, ACE, and Goods_coverage. All indices were calculated using QIIME (Version 1.9.1). Dilution curves, rank abundance curves, and species accumulation curves were generated and displayed with R software (Version 2.15.3). A t-test was used to determine the differences between the alpha diversities of index groups.

### Beta diversity (β-diversity)

Beta diversity analysis was used to evaluate the differences in samples relative to species complexity. β-diversity for both weighted and unweighted Unifrac was calculated using QIIME software (Version 1.9.1). Principal Coordinate Analysis (PCoA) was performed to obtain principal coordinates from complex, multidimensional data. A distance matrix based on Bray–Curtis dissimilarity among samples was obtained before being transformed into a new set of orthogonal axes, by which the maximum variation factor was demonstrated by the first principal coordinate, the second maximum was demonstrated by the second principal coordinate, and so forth. The PCoA was displayed using the WGCNA package, stat package, and ggplot2 package in R software (Version 2.15.3). Unweighted Pair-Group Method with Arithmetic Mean (UPGMA) clustering was performed using QIIME software (Version 1.9.1) as a form of hierarchical clustering to interpret the distance matrix using average linkage. Difference analysis of the beta diversity index between groups was performed using R software, and the test of significance was performed using t-test and Wilcoxon rank-sum test. LEfSe analysis was performed using LEfSe software with the LDA score set to 4. The R vegan package was used to analyze the Anosim, Amova, and Adonis. Species analysis with significant differences between groups was performed using R software for the t-test.

### PCR, sequencing, and phylogenetic analysis

Specific primers were used for *Ureaplasma diversum* identification [31]. Target fragments (215 bp) were cloned and sequenced by Thermo Fisher Co., Ltd. (Shanghai, China). A phylogenetic tree for the 16S rRNA of *Ureaplasma* was obtained automatically using the maximum likelihood approach in MEGA7 (Philadelphia, PA, USA) [32].

## Results

### Diseased pigs displayed more severe symptoms and lung lesions than healthy pigs

**Figure-1:**
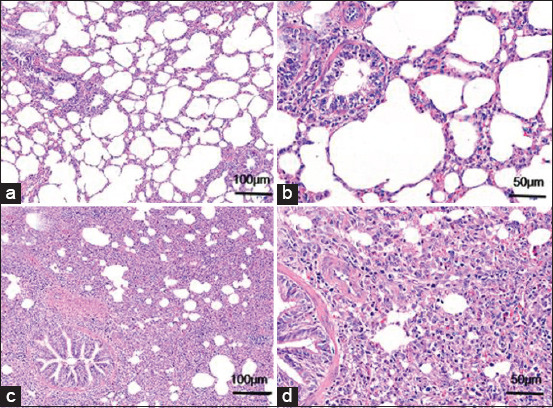
HE staining of lung tissue lesions in experimental pigs. (a and c) Lung tissue lesions of healthy and diseased pigs, respectively, at 100 µm; (b and d) lung tissue lesions of healthy and diseased pigs, respectively, at 50 µm.

All pigs were euthanized for observation of histopathological changes. The histological appearance and sections of the healthy piglets were normal, but the organic appearance of the diseased groups showed the following signs: Mesenteric congestion, pleural effusion and pulmonary adhesion, general lymphadenectasis and hemorrhage (especially in inguinal, mesenteric, and hilar lymph nodes), and the presence of foam-like substances in the trachea (Figure-S1). Lung tissue sections stained with HE were observed under light microscopy. Figure-1a and b show the clear layers in the lung structures of the control animals. Figure-1c and d show the appearance of the lung tissues of pigs with interstitial pneumonia and shows the infiltration of inflammatory cells (neutrophils, lymphocytes, and plasma cells), alveolar wall thickening, and visible necrotic cells.

**Figure-S1:**
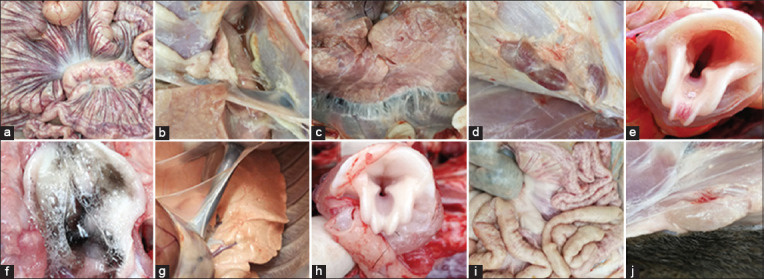
The histopathological changes in experimental pigs (a-f: diseased pigs; g-j: healthy pigs). (a) Mesenteric congestion and enlargement of intestinal lymph nodes. (b and c) Pleural effusion and pulmonary adhesion. (d) Enlargement and congestion of inguinal lymph nodes. (e-f) Foam-like substances present in trachea. (g) Histological appearances of lung, trachea, mesenteric congestion, and intestinal and inguinal lymph nodes were normal.

### Sequence data analysis

**Figure-2:**
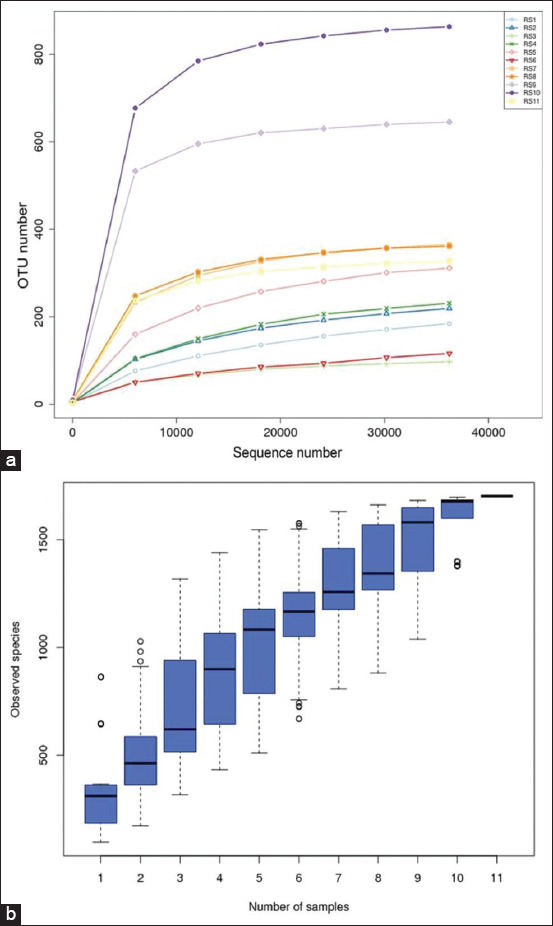
Relationship among sequencing data, sample size, and species diversity. Different samples are represented by curves of different colors. (a) Dilution curve. (b) Species accumulation box chart.

16S rRNA sequencing was performed on the V3-V4 regions of alveolar lavage fluid samples from eleven 4-week-old piglets. After filtering chimeric sequences and mismatches, 818,059 valid sequences were obtained, and 1707 different OTUs were generated at a 97% similarity level, with an average of 374 OTUs per sample (Figure-S2). The dilution curve and species accumulation box chart indicated that the sample and sequencing data volume of this experiment were sufficient to meet the requirements of the downstream analysis of pig lung microbial diversity (Figure-2a and b).

**Figure-S2:**
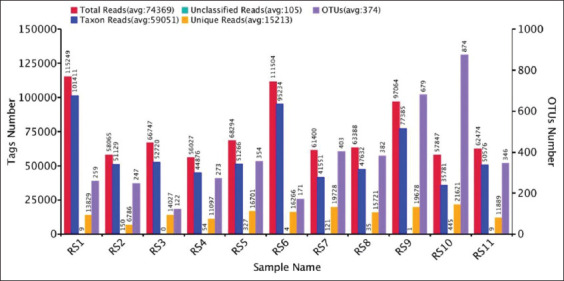
Statistical histogram of reads alignment for all samples.

### Alpha diversity analysis between infected and healthy lungs

Alpha diversity was used to understand the richness and diversity of the microbial communities in the samples, which were counted based on 97% identity thresholds (Table-1, cutoff = 36,226). An average of 611 microorganisms was annotated in the healthy group, but only 235 were annotated in the diseased group, a significant difference (p<0.05). However, no significant differences in the other α-diversity indexes were observed. The results showed that the richness and diversity of microbial species in diseased piglets were lower than those observed for healthy piglets, possibly due to pathogenic bacteria becoming dominant in the lung environment after invading the respiratory tract and destroying the natural microbial balance of the respiratory system.

**Table-1:** Alpha diversity indices of lung microbial community in healthy piglets and piglets with respiratory disease.

Group name	Infected	Control
Observed_species	235.00±103.25*	611.00±269.55
Shannon	2.91±1.05	5.66±2.35
Simpson	0.70±0.15	0.85±0.21
Chao1	267.74±96.74	626.13±271.55
ACE	279.91±90.42	622.05±270.17
Goods_coverage	1.00±0.00	1.00±0.00

Swine respiratory disease decreased the microbial diversity in piglets.

* Significantly different with p<0.05; values are presented as mean±SD

### Changes in taxonomic composition and distribution of swine lung microbiota in response to respiratory disease

**Figure-3:**
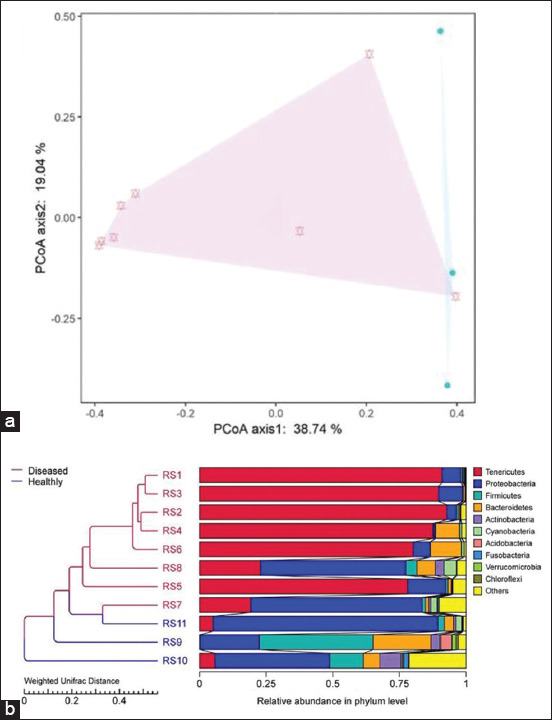
β-diversity analysis. (a) PCoA analysis. The red symbols indicate diseased piglets and the blue symbols indicate healthy piglets. (b) UPGMA clustering tree. The left side is the UPGMA clustering tree structure, and the right side is the relative abundance distribution of the species at the phylum level. Red lines and letters indicate diseased piglets and blue lines and letters indicate healthy piglets.

In all, 28 phyla, 47 classes, 105 orders, 184 families, and 404 genera were annotated in all OTUs. Anosim analysis (R>0, p<0.05) and Amova analysis (p<0.05) verified that the bacterial composition and distribution may not be homogeneous between diseased and healthy samples. To compare the microbial community composition of different samples, PCoA analysis (Figure-3a), based on Bray–Curtis dissimilarity, was employed and showed that all samples were clustered into two categories according to illness and health, which was supported by UPGMA (Figure-3b). The dispersion of the intercontrol group was higher than that of the interinfected group, possibly due to its higher volume.

**Figure-4:**
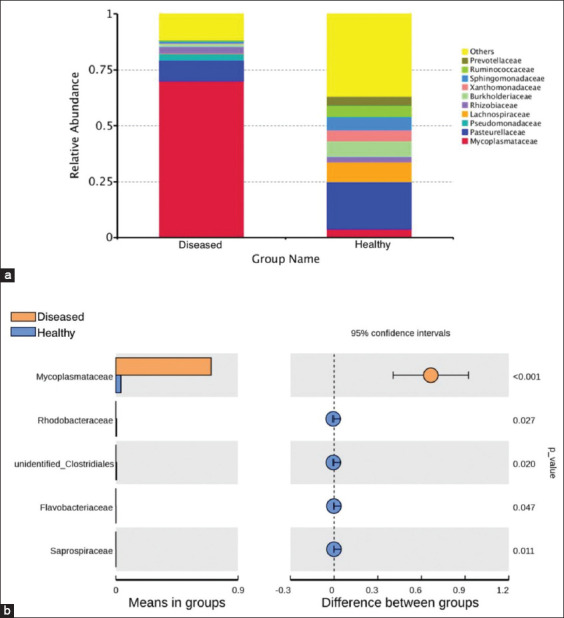
Species annotation results of family level for different groups and t-test. (a) The abundance distribution of the top 10 species in each group. *Mycoplasmataceae* was the most abundant species in ill piglets. (b) t-test. *Mycoplasmataceae* had significantly higher abundance in ill piglets than in healthy piglets (p<0.01).

**Figure-5:**
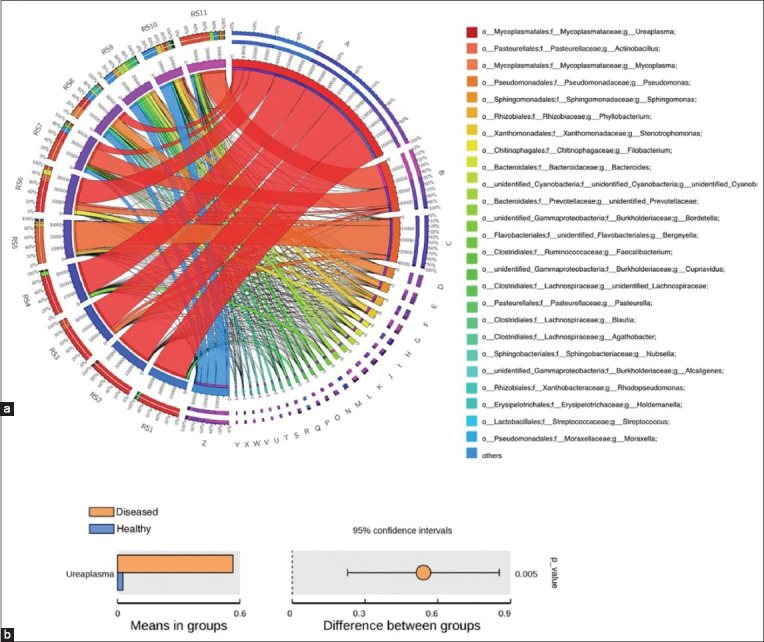
Circos graph of genus level for different samples and t-test. (a) The abundance distribution of the top 26 species in each sample. (b) t-test. *Ureaplasma* had significantly higher abundance in ill piglets than in healthy piglets (p<0.01).

Further analysis was conducted on the taxonomic distribution of swine lung microbiota in response to respiratory disease. At the family level, *Pasteurellaceae* (21.08%), *Lachnospiraceae* (8.79%), and *Burkholderiaceae* (6.65%) were dominant in the healthy piglets, while *Pasteurellaceae* (8.94%) and *Mycoplasmataceae* (70.19%) were the most abundant families in ill piglets. *Mycoplasmataceae*, in particular, was significantly higher in diseased piglets than in healthy piglets (p<0.01) (Figure-4a and b). At the genus level, the dominant bacteria in the healthy group were *Actinobacillus* (20.89%), *Sphingomonas* (5.59%), and *Stenotrophomonas* (5.14%). The dominant bacteria in the diseased group were *Mycoplasma* (13.53%) and *Ureaplasma*, which accounted for 56.66% of the total in diseased piglets but only 2.59% in healthy piglets, a significant difference (p<0.01) (Figure-5a and b). The microbiota of individual piglets and their relative abundance at the phylum (Figure-S3), class (Figure-S4), and order (Figure-S5) levels are shown in the supplementary materials. In ill piglets, the taxonomic composition was relatively simple, but the abundance of some species was relatively high, while in healthy piglets, the opposite trend was observed. It was demonstrated that changes in the swine respiratory microbial balance during respiratory infection were mainly in the abundance of bacteria rather than the composition.

**Figure-S3:**
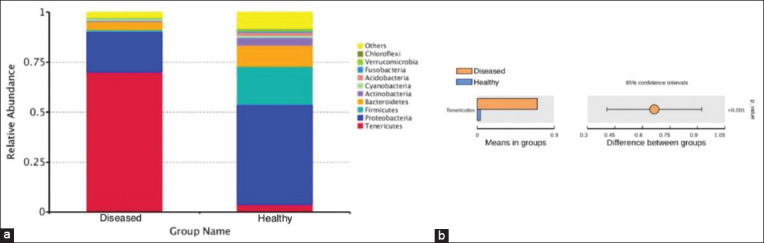
Species annotation results of phylum level for different groups and t-test. (a) The abundance distribution of the top 10 species in each group. *Tenericutes* was the most abundant phylum in ill piglets. (b) t-test. *Tenericutes* had significantly higher abundance in ill piglets than in healthy piglets (p<0.01).

**Figure-S4:**
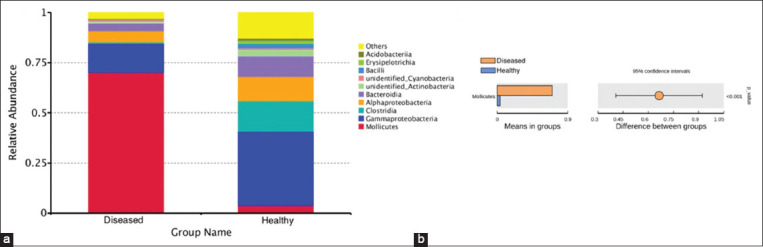
Species annotation results of class level for different groups and t-test. (a) The abundance distribution of the top 10 species in each group. *Mollicutes* was the most abundant class in ill piglets. (b) t-test. *Mollicutes* had significantly higher abundance in ill piglets than in healthy piglets (p<0.01).

**Figure-S5:**
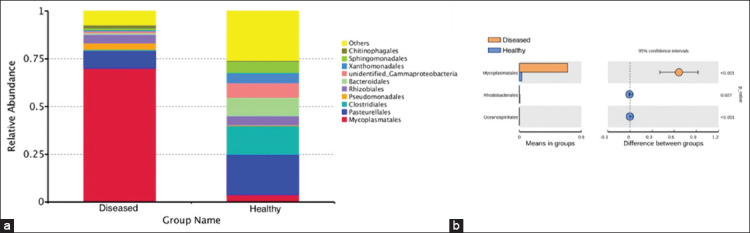
Species annotation results of order level for different groups and t-test. (a) The abundance distribution of the top 10 species in each group. *Mycoplasmatales* was the most abundant order in ill piglets. (b) t-test. *Mycoplasmatales* had significantly higher abundance in ill piglets than in healthy piglets (p<0.01).

### Core flora of Kele piglets with respiratory diseases

**Figure-6:**
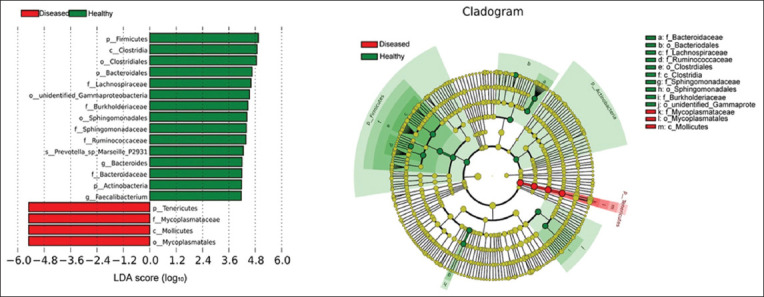
LDA value distribution histogram and evolutionary branch diagram. The histogram for the distribution of LDA values shows species with LDA scores of log_10_ >4. In the evolutionary branch diagram, the circle radiating from the inside to the outside represents the classification level from phylum to genus. Each small circle at the different classification levels represents a classification at that level, and the diameter of the small circle is proportional to the relative abundance. Species with no significant differences are colored yellow.

LEfSe analysis was used to identify the flora of statistical significance by comparing the annotated species between diseased and healthy groups. The characteristic bacteria present in the diseased group included *Tenericutes*, *Mollicutes*, *Mycoplasmatales*, and *Mycoplasmataceae* (LDA score: log_10_ >4.0). However, in healthy piglets, 14 landmark microorganisms were observed, including *Firmicutes* and *Clostridia* (Figure-6). The relative abundance of these organisms in diseased piglets compared to healthy piglets differed significantly (Wilcoxon rank-sum test, p<0.01). LEfSe analysis also indicated that the existence of *Mycoplasmataceae* was sufficient to indicate the occurrence of clinical respiratory disease, but due to the specificity of vaccines and antibiotics, specific genera (*Ureaplasma*, *Mycoplasma*, or both) require verification before targeted treatment and immunization is administered.

**Figure-7:**
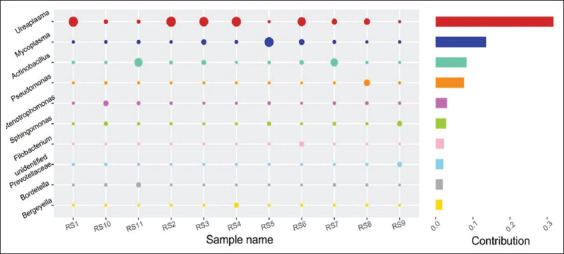
The analysis of microorganisms’ contribution. The vertical axis represents the genus; the horizontal axis represents the samples; the size of the circle represents the relative abundance of the species; “Contribution” is the contribution of the species to the difference observed between the two groups.

Based on the results, we investigated which genera of bacteria were responsible for the observed microbial community shifts in Kele piglets. The analysis indicated that the contributing microorganisms included *Ureaplasma*, *Mycoplasma*, and *Actinobacillus* (Figure-7), and there was a significant correlation between *Ureaplasma* and the infected group (R^2^=0.628, p<0.05). These analyses revealed that the beneficial bacteria in the lungs of piglets with respiratory disease were reduced, and pathogenic genera (mainly *Mycoplasmataceae*) were dominant. The presence of *Ureaplasma* in the diseased piglet lungs indicates that *Ureaplasma* may be involved in the respiratory disease of local pigs in China, in addition to known *Mycoplasma*.

### Phylogenetic tree analysis of *Ureaplasma*

**Figure-8:**
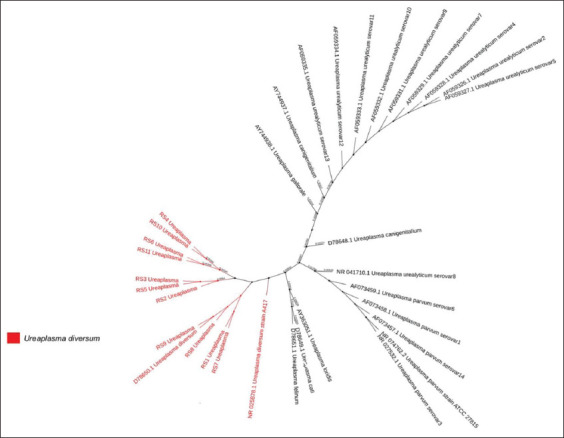
Molecular phylogenetic analysis based on 16S rRNA partial nucleotide sequences of species of the genus *Ureaplasma*.

*M. hyopneumoniae* has been intensively studied as a pathogenic pathogen involved in swine respiratory disease. However, it is not clear which species of *Ureaplasma* plays a potential role in swine respiratory disease. To elucidate this, the OTU sequences clustered to *Ureaplasma* in our results were aligned with the public 16S rRNA genes of bacterial species, and they were found to be most similar to *U. diversum*, an opportunistic pathogen [33]. To further validate the presence of *U. diversum* in infected piglets, we used PCR and sequencing. Target fragments were obtained from experimental piglets using specific primers that were cloned and sequenced. A phylogenetic tree was obtained through comparison with 16S rRNA sequences of *U. diversum*, *Ureaplasma canigenitalium*, *Ureaplasma parvum*, *Ureaplasma urealyticum*, *Ureaplasma gallorale*, *Ureaplasma felinum*, and *Ureaplasma loridis* (Figure-8) and confirmed that the genetic distance between *Ureaplasma* from Kele piglets and *U. diversum* was closest. *U. diversum* may influence the swine respiratory system under natural conditions, potentially explaining the poor efficacy of current vaccination and antimicrobial treatments against bacterial respiratory tract diseases in pigs. The pathogenicity and function of *U. diversum* in pigs requires further study.

## Discussion

In this study, the microbiomes of the lungs of piglets suffering from respiratory disease were characterized. The diversity of the microbial community of ill piglets was markedly lower than that of healthy piglets (p<0.05). *Ureaplasma* and *Mycoplasma* were dominant in the flora of the diseased group, whereas *Actinobacillus*, *Sphingomonas*, and *Stenotrophomonas* were dominant in the healthy group. The combined intensive study of *Ureaplasma* spp. that we isolated, by cluster analysis with published *Ureaplasma* sequences, showed that our *Ureaplasma* was similar to *U. diversum*, a potential pathogen that is known to cause respiratory diseases in livestock.

Overall, the dominant microorganisms at the phylum level in healthy pig lungs were *Proteobacteria*, *Firmicutes*, and *Bacteroidetes*, which are similar to the lung microorganisms present in humans [12,34], mice [35,36], and cattle [21,37]. This finding indicates that the composition of lung microorganisms in healthy individuals of different species is comparable. When swine respiratory disease occurs, the microbiome can change. In this study, we found that *Mycoplasmataceae* (70.19%) was dominant in the lungs of pigs suffering from respiratory disease, which was consistent with the microorganisms found in the lungs of foreign breeds infected with endemic pneumonia [24] and comparable to studies on the microorganisms of the lungs of crossbred pigs [25]. However, at the genus level, *Ureaplasma* (56.66%) and *Mycoplasma* (13.53%) were the two major determinants of clinically diseased local Chinese pig lungs, while *Mycoplasma* (13.0%), *Ureaplasma* (9.2%), *Phyllobacterium* (5.3%), *Sphingobium* (3.2%), and *Haemophilus* (1.8%) were the major organisms in crossbred pig lungs, and *Mycoplasma* had the highest load in foreign breeds. The microbial community in the lungs of pigs suffering from respiratory disease varies between breed, district, and toxicity treatments. Our results emphasize that most swine respiratory diseases are complex infectious diseases caused by multiple pathogens.

During respiratory infections, the relationship between the swine lung microbiome and the host immune response contributes to the clinical manifestations of disease in pigs [38]. In this study, core bacterial analysis of swine respiratory disease showed that *Mycoplasma* and *Ureaplasma* were the two core bacterial genera, with *Ureaplasma* being the most abundant. *Ureaplasma* was first discovered in the bovine reproductive tract [39] and 11% of clinically healthy cows have *Ureaplasma* in their respiratory system [40]; however, a load of *Ureaplasma* in diseased animals is higher than in healthy animals. *Ureaplasma* has also been identified in the respiratory tracts of pigs in Cuba [41] and Canada [42] using PCR. In our research, the *Ureaplasma* phylogenetic tree analysis showed that *Ureaplasma* we isolated was close to *U. diversum*, which was consistent with the previous studies using high-throughput sequencing [43]. Although *U. diversum* was detected in both healthy and sick pigs, its load was significantly different between these two groups (p<0.01), suggesting that *U. diversum* may influence the respiratory system of Kele pigs as a conditional pathogen, or the possibility that we detected different serotyping bacteria in diseased and healthy pigs. For corresponding prevention and treatment plans, culturing, serotyping, and pathogenicity trials should be conducted in further investigations of *U. diversum* to determine whether it causes or aggravates respiratory disease in pigs.

## Conclusion

This study has shown that diseased pigs display more severe symptoms and more lung lesions than healthy pigs. We have also shown that the microbial species and structure of piglets’ lungs were changed during respiratory tract disease. The finding of *Ureaplasma* suggests that besides known pathogens, such as *Mycoplasma* and *Actinobacillus*, other pathogens exist in the lungs of pigs during respiratory diseases and may provide a basis for clinical treatment.

## Authors’ Contributions

KS conceived and designed the study. JW, CZ, CD, and LZ collected and extracted DNA from all samples used in this study. JZ and XZ wrote and revised the manuscript. JZ and KS performed data analysis. All authors read, edited, and approved the final manuscript.
